# Uncovering Anti-Melanoma Mechanisms of *Bambusa stenostachya* Leaf Compounds via Network Pharmacology and Molecular Docking

**DOI:** 10.3390/ijms26136120

**Published:** 2025-06-25

**Authors:** Gen Maxxine C. Darilag, Hsuan-Chieh Liu, Cheng-Yang Hsieh, Lemmuel L. Tayo, Nicholas Dale D. Talubo, Shu-Ching Yang, Ching-Hui Chang, Ying-Pin Huang, Shih-Chi Lee, Yung-Chuan Liu, Po-Wei Tsai

**Affiliations:** 1 School of Chemical, Biological, and Materials Engineering and Sciences, Mapúa University, Metro Manila 1002, Philippines; gmcdarilag@mymail.mapua.edu.ph (G.M.C.D.); nddtalubo@mymail.mapua.edu.ph (N.D.D.T.); 2 Department of Chemical Engineering, National Chung Hsing University, Taichung 402, Taiwan; sjliu@itri.org.tw; 3 Biomass Materials Technology Department, Agri-Industrial Systems Technology Division, Central Region Campus, Industrial Technology Research Institute, Nantou 540, Taiwan; itri526822@itri.org.tw (S.-C.Y.); chinghui@itri.org.tw (C.-H.C.); yphuang@itri.org.tw (Y.-P.H.); hylaman@itri.org.tw (S.-C.L.); 4 Department of Chemical and Materials Engineering, National I-Lan University, I-Lan 260, Taiwan; d339108001@tmu.edu.tw; 5 Department of Food Science, National Taiwan Ocean University, Keelung 202, Taiwan; 6 Department of Biology, School of Health Sciences, Mapúa University, Makati 1200, Philippines; lltayo@mapua.edu.ph

**Keywords:** bamboo leaves, naringin, pathway analysis, skin cancer

## Abstract

Skin cancer, particularly melanoma, remains a major public health concern due to its high mortality rate. Current treatment options, including chemotherapy with dacarbazine and doxorubicin, have shown limited efficacy, achieving only a 20% objective response rate over six months, along with severe side effects such as cardiotoxicity. Given these limitations, there is a growing interest in herbal medicine as a source of novel anticancer compounds. *Bambusa stenostachya*, a bamboo species native to Taiwan, was investigated for its potential anti-melanoma properties using network pharmacology and molecular docking. LC-MS analysis identified seven bioactive compounds, including quinic acid and isovitexin, which satisfied Lipinski’s drug-likeness criteria. Among the seven bioactive compounds identified, five belong to the flavonoid family, while two are classified as phenolic compounds that modulate signaling pathways related to cancer and exhibit antioxidant activity, respectively. Through pathway enrichment analysis, four key melanoma-associated genes (PIM1, MEK1, CDK2, and PDK1) were identified as potential therapeutic targets. Ensemble docking results demonstrated that naringin-7-rhamnoglucoside exhibited the highest binding affinity (−6.30 kcal/mol) with phosphoinositide-dependent kinase-1, surpassing the affinities of standard chemotherapeutic agents. Additionally, the average docking scores for naringin-7-rhamnoglucoside and the remaining three proteins were as follows: PIM1 (−5.92), MEK1 (−6.07), and CDK2 (−5.26). These findings suggest that the bioactive compounds in *B. stenostachya* may play a crucial role in inhibiting melanoma progression by modulating metabolic and signaling pathways. Further in vitro and in vivo studies are necessary to validate these computational findings and explore the potential of *B. stenostachya* as a complementary therapeutic agent for melanoma.

## 1. Introduction

One of the prevailing diseases up until now is skin cancer. This type of cancer involves the abnormal growth of skin cells which is usually caused by sun exposure. One of the most dangerous types of skin cancer is melanoma which may appear as a spot or any discoloration in the skin. Its treatment may vary depending on the severity of the cancer and how fast it spreads throughout the body [1]. The formation of melanoma starts from UV radiation exposure which promotes skin cancer by damaging the DNA including the p53 gene—a tumor suppressor gene—thus leading to carcinogenesis [2]. Studies have shown that existing basal p53 protein-expressing cells that are dispersed throughout the basal layer may represent mutant melanocytes and wild-type p53 in G1-S arrest as a result of DNA damage caused by UV exposure.

As of 2022, an estimated 330,000 people had been recorded to be diagnosed with melanoma, while 60,000 deaths were recorded worldwide [3]. Existing treatments for melanoma have long been established; some of these include surgery, immunotherapy, targeted therapy, radiation therapy, and chemotherapy [4]. In chemotherapy, dacarbazine and doxorubicin are often used as treatments for metastatic melanoma. Dacarbazine is the only drug that is approved by the FDA to treat melanoma which can achieve about 20% of the objective response rate in a 6-month period. Despite the possible success in melanoma treatment using dacarbazine, the drug has limited efficacy as it has not yet provided a good survival benefit regarding metastatic melanoma [5]. Doxorubicin, on the other hand, has also been used to treat melanoma by means of chemotherapy and is often combined with other therapies. A study by Lima et al. in 2023 [6] highlighted the efficiency of the compound which was delivered by a nanoparticle drug in order to enhance its antitumor activity. However, several studies have shown that the compound can cause cardiotoxicity [6].

Nowadays, the use of herbal medicines has been greatly recognized due to the wide range of bioactive compounds with varying characteristics [7]. Several plants have long been used as traditional medicines which are now subjected to analysis for the possibility of becoming a drug [8]. Some of these traditional medicines include traditional Chinese medicine (TCM), Japanese traditional medicine, and Indian traditional medicine [9]. Under TCM are the Taiwanese herbs which are originally foreign but have been “naturalized” in the country. One of the unique plant species that lies in the land of southwestern Taiwan is *bambusa stenostachya* (thorny bamboo). This plant is known to grow healthily despite any conditions including high salinity and poor soil [10]. Moreover, several studies have shown evidence of bamboo having inhibitory effects on melanin synthesis, as well as antioxidant and anti-melanogenic activities [11,12].

To expound the use of the bamboo plant, its bioactive compounds will be subjected to analysis regarding it capability of treating skin cancer. Active compounds from *B stenostachya* were identified using LCMS. It is also worth mentioning that a previous study by Chang et al. in 2024 highlighted the binding potentials of flavonoid compounds from skullcapflavone II (SKII) against the oncogenic proteins of melanoma [13]. The identified oncogenic proteins include the proto-oncogene serine/threonine protein kinase, a mitogen-activated protein kinase, cyclin-dependent kinase 2, and phosphoinositide-dependent kinase-1. Building upon the identification of key oncogenic targets by the previous study, this study focuses on the analysis of those proteins to better understand their therapeutic relevance in melanoma through network pharmacology. By integrating computational target prediction, as well as analyzing protein–protein interactions and pathway enrichment analysis, this study aims to identify novel compound and protein associations to elucidate the underlying mechanism on which *B. stenostachya* molecules may exert anti-tumorigenic effects.

## 2. Results

### 2.1. LCMS Analysis

In our previous study, it was reported that the results of the LC-MS analysis revealed the presence of seven compounds (Table 1) [14]. The LC-MS results identified cyclitol, phenolic acids, and flavonoids. The detected classes of compounds in *B. stenostachya* may play significant roles in the construction of network pharmacology for melanoma due to the diverse properties of each group, which can aid in the regulation of the disease.

### 2.2. Bioactive Compounds from B. stenostachya

The screening of the seven bioactive compounds from *B. stenostachya* revealed that there were only two compounds that were qualified for being a drug which were quinic acid and isovitexin—satisfying the Lipinski’s rule of five with 0 and 1 violations, respectively (Table 2). Here, isovitexin had seven hydrogen bond donors which failed to satisfy the cutoff of <5 H bond donors. On the other hand, the five remaining compounds, which were chlorogenic acid, homoorientin, orientin, vitexin, and narinigin-7-rhamnoglucoside, were not able to satisfy the Lipinski categories, as well as the bioavailability and oral bioavailability thresholds. 

### 2.3. Predicted Target Genes of B. stenostachya and Related Genes of Melanoma

**Figure 1:**
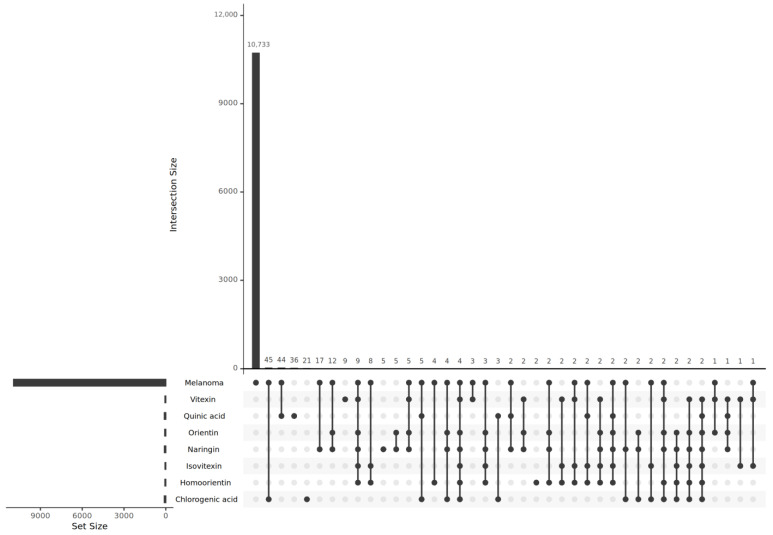
UpSet plot presenting an intersectional analysis of predicted compound target proteins from SwissTarget, Pharmmapper, and SuperPred, alongside known melanoma-associated proteins curated by GeneCards.

There was a total of 193 proteins that overlapped as targets of the compounds and as potentially relevant genes to melanoma. As observed from Figure 1, chlorogenic acid appears to have the most targeted proteins that can potentially affect melanoma. This is followed by quinic acid and naringin. To ensure clinical relevance, well-known melanoma-related proteins from previous studies were included for this study [13]. These proteins include the proto-oncogene serine/threonine protein kinase (PIM1), a mitogen-activated protein kinase (MEK1), cyclin-dependent kinase 2 (CDK2), and phosphoinositide-dependent kinase 1 (PDK1).

### 2.4. Pathway and Process Enrichment Analysis

**Figure 2:**
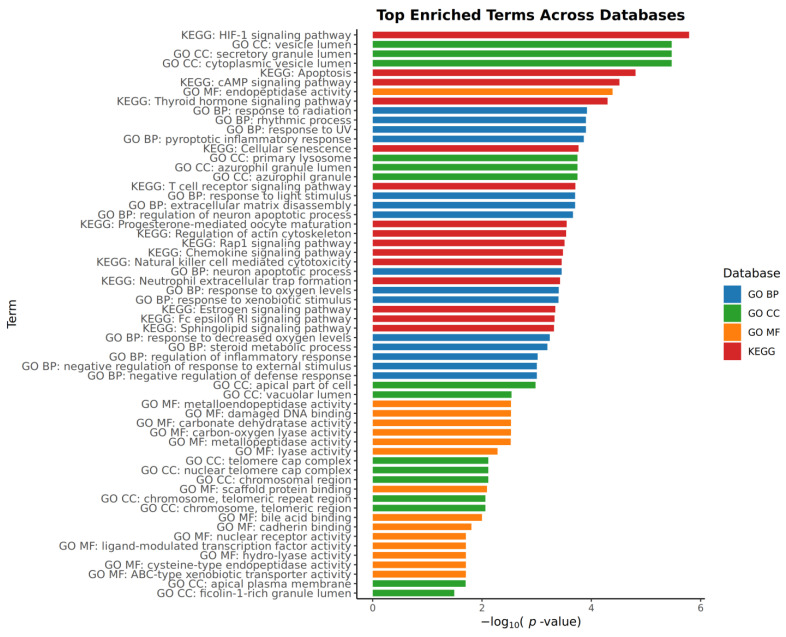
Bar graph of top enriched terms across databases. The databases include KEGG, the GO Biological Process, GO Cellular Components, and the GO Molecular Function.

Pathway enrichment analysis was performed on the combined targets of all the test compounds and melanoma genes. In Figure 2, a bar plot was used to plot the results. The top significantly enriched KEGG pathways included those related to metabolic regulation and cancer progression, such as ‘Central carbon metabolism in cancer’ (hsa05230) and ‘Chemical carcinogenesis—receptor activation’ (hsa05207). Key signaling pathways such as the ‘HIF-1 signaling pathway’ (hsa04066) and ‘PD-L1 expression and PD-1 checkpoint pathway in cancer’ (hsa05235) were also identified. Additionally, pathways like ‘Apoptosis’ (hsa04210) and ‘Insulin resistance’ (hsa04931) highlighted cell death and metabolic mechanisms, while ‘Prostate cancer’ (hsa05215) indicated possible cross-cancer applications.

For the Gene Ontology Cellular Component database, the results indicate that significant groups of genes were found in the lumen. More specifically they are seen in the secretory granule lumen (GO:0034774), cytoplasmic vesicle lumen (GO:0060205), and vesicle lumen (GO:0031983). Biologically, the enrichment shows that the targeted genes are potentially involved in response to radiation (GO:0009314), more specifically response to UV (GO:0009411). The two terms are the top two enriched for the GO Biological Process. Lastly for the GO Molecular Function, the presence of endopeptidase activity (GO:0004175), damaged DNA binding (GO:0003684), and dehydratase activity (GO:0004089) was noted to be the top three most enriched.

Notably, several of the enriched KEGG pathways are highly relevant to melanoma pathogenesis. Although not the top ranked in the bar plot, the pathways deserve emphasis due to their critical role in melanoma cell proliferation and treatment conditions. These pathways include the MAPK and RAS signaling pathways, the PI3K-Akt signaling pathway, and MicroRNAs in cancer, with *p*-values lower than 0.05. Together, these findings validate the biological relevance of the added proteins, namely PIM1, MEK1, CDK2, and PDK1, in the central pathways of melanoma.

### 2.5. Ensemble Docking Results

**Figure 3:**
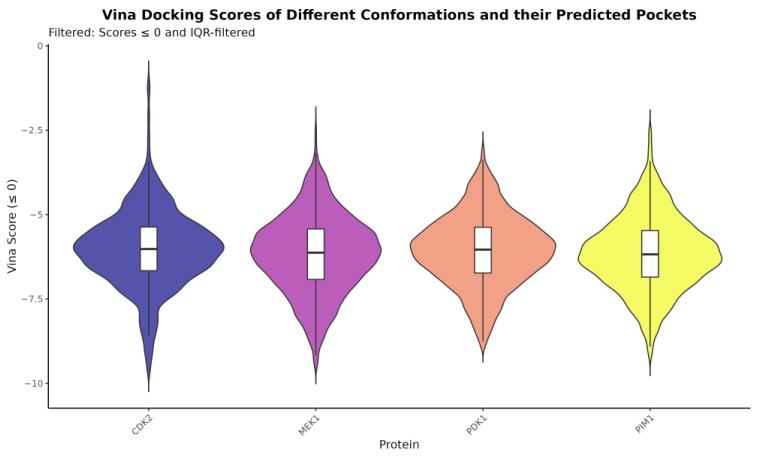
Violin plots depicting Vina docking scores across different protein conformations and their predicted binding pockets. Only pockets where the test compounds exhibited successful binding were included. Outliers were removed using interquartile range (IQR).

**Figure 4:**
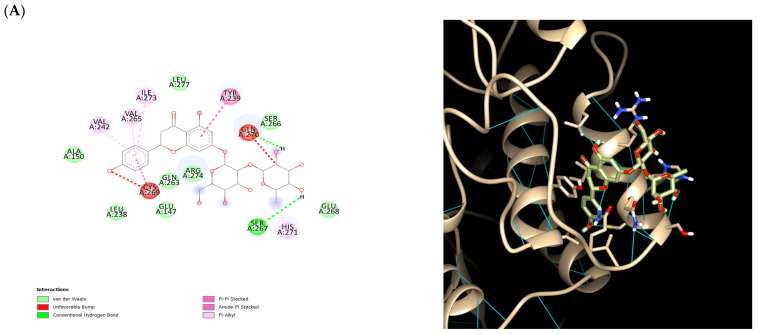

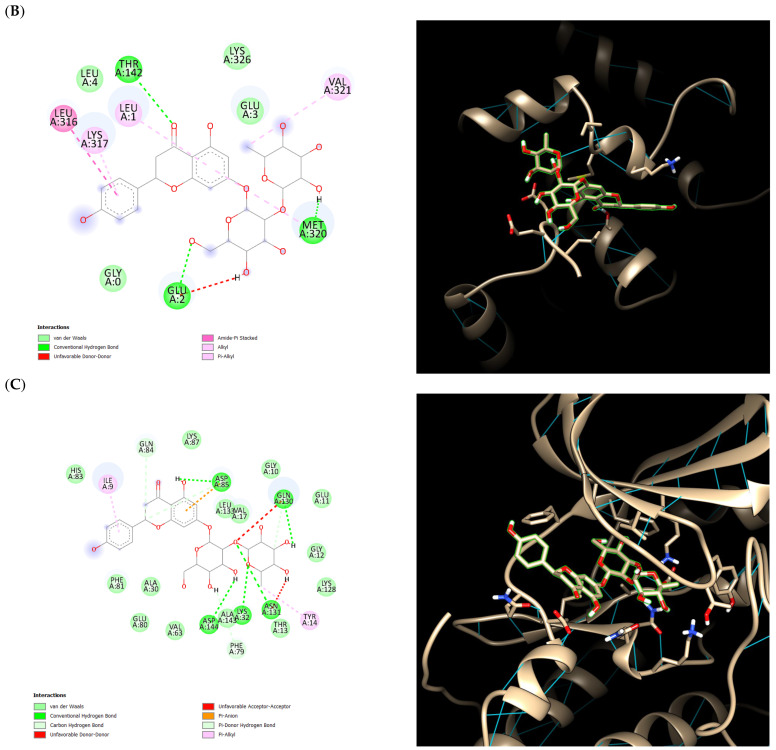

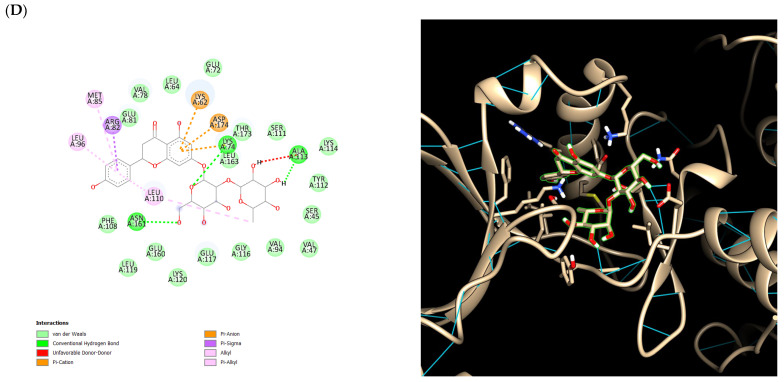
(**A**) Naringin-7-rhamnoglucoside and proto-oncogene serine/threonine protein kinase complex. (**B**) Isovitexin and mitogen-activated protein kinase complex. (**C**) Naringin-7-rhamnoglucoside and cyclin-dependent kinase 2 complex. (**D**) Naringin-7-rhamnoglucoside.

To further validate the results, the bioactive compounds chlorogenic acid, quinic acid, orientin, homoorientin, vitexin, isovitexin, and naringin-7-rhamnoglucoside were docked with the previously identified melanoma protein targets which were the proto-oncogene serine/threonine protein kinase, a mitogen-activated protein kinase, cyclin-dependent kinase 2, and phosphoinositide-dependent kinase-1. Table 3 details the consensus scores achieved by each protein–ligand pair. With a total of nine docking poses, multiple pocket predictions, and 50 different conformations for each protein, naringin-7-rhamnoglucoside consistently ranked first for the average, median, and corrected mean scores for all four melanoma related proteins. Figure 3 illustrates the distribution of Vina scores for the predicted binding pockets across protein conformations. The distributions are approximately symmetrical around the mean, suggesting consistent ligand-binding stability. Furthermore, the most favorable protein–ligand complexes for naringin-7-rhamnoglucoside are highlighted in Figure 4. It is significant that the average docking scores for naringin-7-rhamnoglucoside and the melanoma-related proteins were as follows: PIM1 (−5.91897), MEK1 (−6.07004), CDK2 (−5.26133), and PDK1 (−6.29952). The negative binding energies suggest that the interactions between the ligand and the proteins were spontaneous. Here, docking was performed along with the controls dacarbazine and doxorubicin, which were both used to treat melanoma. The two compounds yielded an average docking score of −3.67363 and −5.21502, respectively. Another point of interest is the intermolecular forces acting upon the complexes. The complexes also displayed unfavorable donor–donor bonds and unfavorable bumps which may need further validation. Despite these results, it is still important to highlight the consistency of the low binding energies of naringin-7-rhamnoglucoside with the melanoma-related proteins. It is also noteworthy that the general intermolecular forces involved in the binding were van der Waals, conventional hydrogen bonding, and carbon–hydrogen bonds.

**Figure 5:**
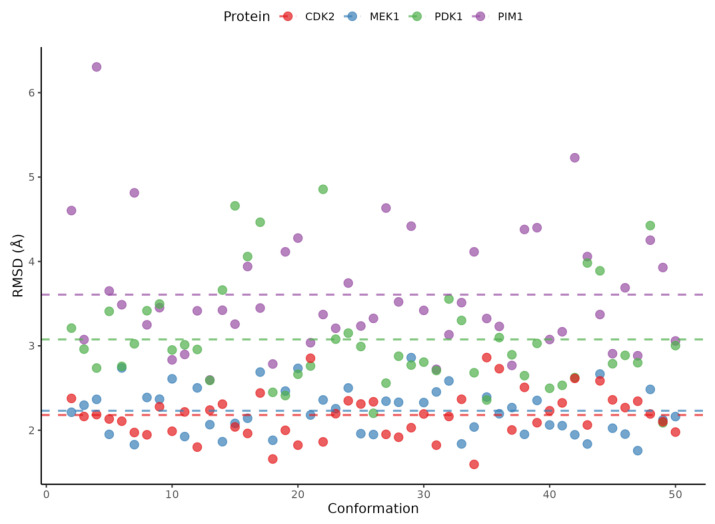
Root Mean Square Deviation (RMSD) of each conformation compared to the original structure. The colored dash lines indicate the calculated mean for the RMSD of each protein.

To conduct ensemble docking, the protein conformations of the four proteins need to be predicted. Figure 5 presents a scatter plot detailing the Root Mean Square Deviation (RMSD) for each generated conformation. The calculated average RMSD values were 3.607 Å for PIM1, 2.231 Å for MEK1, 2.180 Å for CDK2, and 3.076 Å for PDK1. These diverse RMSD values are indicative of varied binding orientations, a consequence of the inherent dynamic nature and flexibility characteristic of these proteins.

## 3. Discussion

Melanoma is a type of skin cancer that is caused by alterations in the melanocytes which are cells that are responsible for giving pigments in the skin—called melanin [15]. Although drugs like dacarbazine and doxorubicin were found to effectively treat melanoma, there are no clear mechanisms on how the drugs produce antitumor activity except by inducing DNA methyl adducts in tumor cells after hepatic metabolism in dacarbazine. On the other hand, it was recently known that doxorubicin can contribute to DNA damage, the production of reactive oxygen species, apoptosis, senescence, and an immunomodulatory role [16,17]. With the occurrence of herbal medicines and their increasing scientific validations, *B. stenostachya* has been subjected to analysis due to its impact on melanoma [18]. In relation to this, several studies have found that some bamboo strains could produce antioxidative and anti-melanogenic activities. For instance, a study by Choi et al. revealed that *Phyllostachis nigra,* a Korean bamboo strain, was capable of downregulating melanin production [11]. Similarly, a study conducted by Ashour et al. discussed the inhibitory effects of *Phyllostachis pubescens* against melanin production as well as cancer cells using a three-dimensional model of the human skin [12].

Through network pharmacology and molecular docking, it was revealed that the bioactive compounds of *B. stenostachya* have the potential to be natural drugs against melanoma. Through literature mining, it was found that the four known melanoma-related proteins PIM1, MEK1, CDK2, and PDK1 can potentially be targets of the bioactive compounds of *B. stenostachya.*

The proto-oncogene, serine/threonine kinase (PIM1) belongs to the Ser/Thr protein kinase family and is overexpressed in hematopoietic malignancies and in prostate cancers. It is known to play a contributing role in cell proliferation and survival, as well as a selective advantage in tumorigenesis [19]. In a study conducted by Tursynbay et al., PIM1 was reported to be an emerging cancer drug target as it is localized in the nucleus and plasma membrane. PIM1 was found to be associated with the drug resistance abilities of cancer cells by interacting with other cancer-related proteins. Additionally, PIM1 was found to be a senescence regulator, an epigenetic dynamics regulator, and a biomarker for prostate cancer [20]. Similarly, mitogen-activated protein kinase 1 (MEK1), also known as MAP2K1, is an essential component of the MAP kinase signal transduction pathway which is involved in many cellular processes including cell proliferation, differentiation, transcription, and regulation [21]. Additionally, according to a study by Mizuno et al., the MAP kinase was identified to be involved in several human malignancies including melanoma [22]. Cyclin-dependent kinase 2 (CDK2) equally plays a crucial role in cancers, especially that of cell cycle regulation. CDK2 is involved in the control of the cell cycle and is essential for meiosis. With its over-activation in cancers, it has been subjected to many studies as a target for cancer therapy [23]. Lastly, phosphoinositide-dependent kinase 1 (PDK1) is another essential Ser/Thr protein that plays a significant role in cell growth and proliferation—making it the ‘master’ kinase as it is capable of activating at least 23 downstream protein kinases that are enriched in multiple signaling pathways [24]. In cancers, this protein was observed to be a regulator of cancer cell proliferation, survival, and metabolism. It was highlighted to be particularly related to the MAPK4 and PI13/AKT pathways by activating AKT at the cell membrane. Moreover, with its activity on other downstream kinases, PDK1 is capable of driving tumor-promoting effects even outside the said pathways [25].

The pathway enrichment analysis revealed the four melanoma-related proteins PIM1, MEK1, CDK2, and PDK1 in several pathways including the pathways related to cancer. Interestingly, MEK1 was found to be enriched in the MAPK and RAS signaling pathways which are the major signaling pathways in melanoma [26,27]. MEK1 is responsible for phosphorylating downstream substrates that ultimately influence gene expression [10]. With sufficient evidence that the protein plays a substantial role in cancer progression, it may imply that MEK1 could be a potential target by the bioactive compounds of *B. stenostachya.* Likewise, MEK1 and CDK2 were found to be enriched in the PI3K-Akt signaling pathway. This pathway is involved in melanoma initiation, progression, and treatment resistance [28]. MEK1 and its pathway are interdependent, whereas the PI3K-Akt signaling pathway serves as a mediator for the MEK1 protein’s ability to suppress apoptosis [29]. In this context, the combined inhibition of the said pathway and protein may suggest a synergistic targeting strategy to treat melanoma. In view of the account that CDK2 is also enriched in the PI3K-Akt signaling pathway, targeting the said protein may enhance antitumor activity. Similarly, due to the fact that CDK2 regulates cell cycle progression through its interaction with other cyclins, it is vulnerable for defects regarding their expression, especially for cancers. The similar interdependence of the aforementioned pathway and CDK2 is linked through the modulation of CDK2 inhibitors, which explains growth factor signaling to cell cycle progression, therefore promoting uncontrolled cell proliferation in cancers [30]. Additionally, the PDK1 protein was found to be enriched in the central carbon metabolism in cancer which is significantly altered in cancer cells to support their rapid proliferation, as well as survival [31]. PDK1 is responsible for the regulation of pyruvate into the tricarboxylic acid (TCA) cycle. The inhibition of the pyruvate dehydrogenase complex (PDH) can divert the pyruvate itself from the TCA cycle to glycolysis which is a key pathway for the Warburg effect in many cancers. In this context, targeting PDK1 may sensitize cancer cells to therapy [32]. Conversely, PIM1 was found to be enriched in several pathways including microRNAs in cancer and acute myeloid leukemia. Although the said pathways are indirectly relevant to melanoma, reports and studies highlight the promising potential of the protein itself to be a target to treat cancer. For instance, a study by Choudhury et al. reported that novel compounds that were able to inhibit PIM1 showed effectiveness and a positive toxicity profile in preclinical studies [32]. It is also worth mentioning that PIM1 regulators include microRNAs, estrogen, inecalcitol, and adenosine triphosphate (ATP) [33].

In light of these findings, it becomes clear that melanoma cells exploit a diverse range of pathways which can be targeted by the bioactive compounds of *B. stenostachya*, particularly PIM1, MEK1, CDK2, and PDK1. These pathways collectively suggest that melanoma can be modulated by pleiotropic targeting to enhance anti-melanoma drugs, as well as directly inhibit the molecules involved in the formation of melanoma. Ultimately, the ability to engage in multi-target drug development underscores the importance of exploring natural products in the context of melanoma and other cancer research.

To further validate the relationship of *B. stenostachya* and melanoma, the bioactive compounds of the plant were docked with the melanoma related proteins which include the proto-oncogene serine/threonine protein kinase, a mitogen-activated protein kinase, cyclin dependent kinase 2, and phosphoinositide-dependent kinase-1 and were compared with two controls. Specifically, ensemble docking was performed in order to consider the dynamic nature of the four melanoma proteins, where 50 different conformations were used. It was revealed that the top complexes involve naringin-7-rhamnoglucoside which is a flavonoid that is usually found in citrus fruits. Despite its low bioavailability, several studies highlighted the importance and potential of the compound in the medicinal context including its anti-inflammatory and antioxidant properties [34]. Furthermore, the compound demonstrated a significantly higher binding affinity compared to the controls for all four targets and exhibited the strongest binding affinity with PDK1. The average binding affinities of naringin-7-rhamnoglucoside for PIM1, MEK1, CDK2, and PDK1 were −5.91897, −6.07004, −5.26133, and −6.29952, respectively. As opposed to the controls, dacarbazine and CDK2 exhibited the highest binding affinity of −5.94061, while the lowest binding affinity for the controls was for the doxorubicin and PDK1 complex—having an average affinity of −3.67363. With these results, naringin-7-rhamnoglucoside poses promising potential as a treatment for melanoma. Since the compound comes from a natural product, it is more biocompatible to the human body and has fewer side effects. Consequently, naringin-7-rhamnoglucoside has a shorter half-life and higher biodegradability compared to other synthetic drugs [35]. These results also imply that naringin-7-rhamnoglucoside could be a potential multi-target drug and can be combined with other anti-tumor drugs to enhance its treatment methods.

## 4. Materials and Methods

### 4.1. Materials and Sample Preparation

The leaves of *B. stenostachya* were collected from the Southern Region Campus of the Industrial Technology Research Institute (ITRI-SRC), Liujia Dist., Tainan City, Taiwan, and were authenticated by Dr. Ching-Hui Chang and Dr. Ying-Pin Huang from ITRI-SRC, Taiwan. A voucher specimen # ITRI-SRC-BSL-001 of *B. stenostachya* was deposited at the Southern Region Campus of ITRI, Taiwan. The leaves of *B. stenostachya* were oven dried at 40 °C for three days. Approximately 50 g of the powdered leaves was extracted using 50% EtOH at 100 °C in a solid/solvent ratio of 1:20. The crude extract was filtered out of the mixture, and the solvent was evaporated under vacuum conditions for freeze-drying.

### 4.2. LCMS Analysis of B. stenostachya Leaf Crude Extract

The compound identification method has been previously described in our published work [14]. Briefly, the extract was analyzed using LC-MS/MS (Nexera X2 coupled with LCMS-8045, Shimadzu, Japan) equipped with an ESI source and a Kromasil C18 column (4.6 × 250 mm). The mobile phases consisted of 0.1% formic acid in water (A) and acetonitrile (B), with a gradient elution at a flow rate of 0.4 mL/min. The analysis was conducted at 25 °C in negative ion mode over an *m*/*z* range of 50–1000 Da [12].

### 4.3. Screening of Bioactive Compounds from B. stenostachya

After the identification of compounds from *B. stenoctahcya,* the compounds were screened based on their physicochemical properties including absorption, distribution, metabolism, and excretion using SwissADME (http://www.swissadme.ch/index.php, accessed on 28 July 2024) [36]. Here, a threshold bioavailability score of >0.55 was set, and an oral bioavailability threshold of >30% was set.

### 4.4. Target Prediction of Bioactive Compounds from B. stenostachya

The target genes of the compounds were predicted using three tools: Pharmmapper, SwissTarget, and SuperPred [37,38,39]. To ensure the homogeneity of the identifiers, all unique UniProt IDs were retrieved and subsequently translated into gene names through the UniProt website (https://www.uniprot.org/id-mapping, accessed on 24 May 2025) [40]. Consequently, any predicted targets without the associated UniProt IDs were excluded. Furthermore, only the top 500 predicted targets were retained from Pharmmapper. For SwissTarget, a filtering criterion of a probability greater than 0 was applied. Finally, SuperPred predictions were filtered to include only those exhibiting probability and model accuracy scores of 60 or higher. Also, well-known melanoma proteins were subjected primarily to the downstream process of this study. These proteins were obtained from a previous study conducted by Chang et al. in 2024 [13].

### 4.5. Pathway Enrichment Analysis

Following this, an UpSet plot was generated to determine the intersection of the individual target gene sets for the seven compounds with the melanoma gene set, which was sourced from the GeneCards website [41]. This process resulted in seven specific intersections. The collective union of these sets created the definitive set of genes hypothesized to be influenced by the bioactive compounds of *B. stenoctahcya*. Subsequently, a pathway enrichment analysis was conducted by employing ClusterProfiler (version 4.16.0) [42]. Four databases were leveraged to enrich the gene set: Gene Ontology (GO) including the Biological Process (BP), Molecular Function (MF), and Cellular Component (CC) through the enrichGO function and the KEGG database through enrichKEGG. Furthermore, the organism for the gene set was explicitly set to ‘hsa’ (*Homo sapiens*).

To further elucidate the functional context of the common target genes, a cutoff adjusted *p*-value of <0.05 was applied to prioritize the biologically relevant annotations [43]. Subsequently, the traced annotations were ranked according to the top 15 most relevant pathways and gene ontology terms. Furthermore, KEGG is the most comprehensive database in terms of species [44].

### 4.6. Molecular Docking Preparation

In order to validate the results from the Network Pharmacology of *B. stenostachya* and melanoma, a blind molecular docking procedure for the seven bioactive compounds of bamboo- and melanoma-related genes was conducted. The structures of the ligands were obtained from PubChem and translated into PDB form using Open Babel (https://openbabel.org/index.html, accessed 24 May 2025) [45] which is a chemical toolbox for most chemical data. Moreover, the structures of the protein targets were obtained from the Protein Data Bank (https://www.rcsb.org/, accessed 24 May 2025) [46]. The consequent protein models including the proto-oncogene serine/threonine protein kinase, a mitogen-activated protein kinase, cyclin-dependent kinase 2, and phosphoinositide-dependent kinase-1, along with their respective resolutions, are displayed in Table 4.

### 4.7. Ensemble Docking

To address the flexibility and dynamic nature of the protein targets, multiple conformations were generated and used for docking. Crucially, all subsequent analyses and tool-based processes for ensemble docking were conducted via the Galaxy Europe platform (https://usegalaxy.eu/, accessed on 25 May 2025), which is a web-based platform that allows for the accessible computational workflow of this study [47]. An in-house script from GitHub Version 3.16.2 was used to generate 50 conformations for each protein (https://github.com/giacomo-janson/sam2, accessed on 25 May 2025) [48]. This script uses the mdCATH-based aSAMt model. Furthermore, a standard temperature of 310 Kelvin was utilized. Afterward, the pdb files of the 4 proteins were prepared using PDBFixer Version 1.8.1 in Galaxy EU. Here, all missing atoms were added, while heterogens were removed. The nonstandard residues were also replaced with standard residues, and the missing residues were added. The pH was set as 7.0, which was also the default acidity for the proteins. Next, the binding pockets of all the proteins were predicted using the fpocket tool from Galaxy EU version 4.0.0+galaxy0. This tool is capable of finding potential binding sites in protein structures by relying on geometric alpha-sphere theory to identify the small-molecule binding pockets [49]. Next, the box parameters of the proteins were defined using RDKit Version 2021.03.05. This tool is specifically used to define the binding site axes for AutoDock Vina. Both the ligands and the receptors were then prepared using the prepare ligand and receptor tools from Galaxy Version 1.5.7+galaxy0. Lastly, with all the prepared files, docking was conducted using AutoDock Vina from Galaxy Version 1.2.3+galaxy0 [50]. The ligand protonation was set to 7.0, while the number of poses was set to 9.

## 5. Conclusions

Overall, this study successfully utilized the combination of network pharmacology and molecular docking to study the compounds and potentials of *B. stenostachya* in the treatment of melanoma. The highly supported results have shown that naringin-7-rhamnoglucoside from *B. stenostachya* could possibly target the genes involved in the progression of melanoma, specifically PIM1, MEK1, CDK2, and PDK1. The pathway enrichment analysis revealed that these proteins are involved in five significant pathways relevant to melanoma. Through gene ontology analysis, the proteins were found to be mostly concentrated as a cellular component in the lumen and in the plasma membrane. Consequently, the proteins were found to function molecularly in catalytic, binding, and transport activities. Lastly, the four proteins were found to be centralized in biological processes such as responses to stimuli, cell death and inflammation, and metabolic processes. This reflects that *B. stenostachya* has the potential to treat and prevent melanoma by means of modulating the said targets and pathways. The results of molecular docking indicate the spontaneous binding activity of all the bioactive compounds on the melanoma-related proteins—suggesting a possible interaction within the molecules and the targets. Additionally, the high affinity of naringin-7-rhamnoglucoside and the proto-oncogene serine/threonine protein kinase compared to the controls dacarbazine and doxorubicin reflects on the possibility that the drugs to be developed have higher efficiency than existing drugs. Moreover, the results of this study are significant to the development of cancer drugs, specifically, for melanoma. Despite the limitation of conducting this study in silico, further experimental research can be performed to study and validate the findings of this study.

## Figures and Tables

**Table 1:** List of identified compounds. PubChem ID from *B. stenostachya* leaf extract.

PubChem ID	Chemical	Molecular Weight (g/mol)	Molecular Formula
6508	Quinic acid	192.17	C_7_H_12_O_6_
1794427	Chlorogenic acid	354.31	C_16_H_18_O_9_
5280441	Vitexin	432.4	C_21_H_20_O_10_
162350	Isovitexin	432.4	C_21_H_20_O_10_
114776	Homoorientin	448.38	C_21_H_20_O_11_
5281675	Orientin	448.4	C_21_H_20_O_11_
442428	Naringin (Naringenin 7-rhamnoglucoside)	580.5	C_27_H_32_O_14_

**Table 2:** Bioactive compounds of *B. stenostachya*.

Molecule	GI Absorption	Bioavailability Score	OB%	Structure
Chlorogenic acid	Low	0.11	13.61	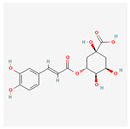
Quinic acid	High	0.56	63.53	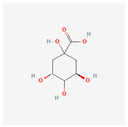
Homoorientin (isoorientin)	Low	0.17	23.3	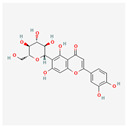
Orientin	Low	0.17	1.79	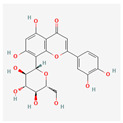
Vitexin	Low	0.55	3.05	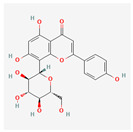
Isovitexin (rutin and ferulic acid)	Low	0.55	31.29	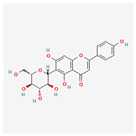
Naringin-7-rhamnoglucoside	Low	0.17	6.92	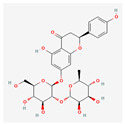

**Table 3:** Summarized consensus scores across binding pockets after ensemble docking.

Protein	Ligand	csMIN	csAVG	csMED	csTRIMMEAN
CDK2	CHLOROGENIC ACID	−9.43000	−4.92246	−5.91150	−5.18689
CDK2	DACARBAZINE	−6.11900	−3.67363	−4.36200	−3.87281
CDK2	DOXORUBICIN	−86.06400	−5.21502	−5.88450	−5.13361
CDK2	HOMOORIENTIN	−9.63400	−4.89919	−5.75800	−5.11512
CDK2	ISOVITEXIN	−9.60300	−4.81899	−5.75450	−5.03151
CDK2	NARINGIN	−10.17400	−5.26133	−6.20200	−5.48337
CDK2	ORIENTIN	−12.12300	−5.05633	−5.98100	−5.26331
CDK2	QUINIC ACID	−8.12300	−4.79348	−5.66450	−5.04931
CDK2	VITEXIN	−10.40400	−4.96989	−5.93700	−5.17481
MEK1	CHLOROGENIC ACID	−8.99900	−5.54884	−6.07900	−5.94549
MEK1	DACARBAZINE	−6.33300	−4.06540	−4.46100	−4.37759
MEK1	DOXORUBICIN	−20.71700	−5.71248	−6.14900	−6.01304
MEK1	HOMOORIENTIN	−10.53400	−5.63281	−6.12200	−5.99234
MEK1	ISOVITEXIN	−11.00200	−5.59132	−6.09000	−5.95523
MEK1	NARINGIN	−15.06100	−6.07004	−6.59300	−6.42142
MEK1	ORIENTIN	−12.31800	−5.77236	−6.25400	−6.15324
MEK1	QUINIC ACID	−7.70700	−5.18088	−5.67900	−5.58443
MEK1	VITEXIN	−10.73900	−5.69276	−6.21200	−6.06392
PDK1	CHLOROGENIC ACID	−10.69600	−5.84483	−6.08850	−6.12630
PDK1	DACARBAZINE	−6.55200	−4.26388	−4.47700	−4.47145
PDK1	DOXORUBICIN	−17.18300	−5.94061	−6.16500	−6.18560
PDK1	HOMOORIENTIN	−9.87300	−5.88804	−6.12450	−6.14669
PDK1	ISOVITEXIN	−9.52400	−5.83747	−6.06300	−6.08772
PDK1	NARINGIN	−10.60100	−6.29952	−6.54000	−6.57658
PDK1	ORIENTIN	−10.18200	−6.00952	−6.27700	−6.26988
PDK1	QUINIC ACID	−8.24700	−5.43671	−5.70000	−5.69214
PDK1	VITEXIN	−10.14200	−5.94927	−6.18250	−6.19803
PIM1	CHLOROGENIC ACID	−9.54600	−5.52084	−6.11100	−5.92045
PIM1	DACARBAZINE	−6.72500	−4.09535	−4.45800	−4.39848
PIM1	DOXORUBICIN	−50.59300	−5.57615	−6.08800	−5.86939
PIM1	HOMOORIENTIN	−10.45500	−5.58319	−6.20750	−5.94528
PIM1	ISOVITEXIN	−10.69600	−5.52624	−6.13400	−5.88888
PIM1	NARINGIN	−17.22900	−5.91897	−6.54600	−6.30309
PIM1	ORIENTIN	−11.47800	−5.60748	−6.28100	−5.98845
PIM1	QUINIC ACID	−8.51000	−5.33340	−5.89500	−5.73615
PIM1	VITEXIN	−10.43800	−5.53094	−6.14600	−5.91048

**Table 4:** Protein models from the Protein Data Bank.

PDB	PDB ID	Protein Name	Resolution
3A99	pdb_00003a99	proto-oncogene serine/threonine-protein kinase	1.60 A
2R3Q	pdb_00002r3q	mitogen-activated protein kinase	1.35 A
5LVO	pdb_00005lvo	cyclin-dependent kinase 2	1.09 A
7B7R	pdb_00007b7r	phosphoinositide-dependent kinase-1	1.70 A

## Data Availability

All data are included in the article.
